# Electrochemical behavior of polypyrrol/AuNP composites deposited by different electrochemical methods: sensing properties towards catechol

**DOI:** 10.3762/bjnano.6.209

**Published:** 2015-10-21

**Authors:** Celia García-Hernández, Cristina García-Cabezón, Cristina Medina-Plaza, Fernando Martín-Pedrosa, Yolanda Blanco, José Antonio de Saja, María Luz Rodríguez-Méndez

**Affiliations:** 1 Department of Inorganic Chemistry, Engineers School, Universidad de Valladolid, 47011 Valladolid, Spain; 2 Department of Materials Science, Engineers School, Universidad de Valladolid, 47011 Valladolid, Spain; 3 Department of Condensed Matter Physics, Faculty of Sciences, Universidad de Valladolid, 47011 Valladolid, Spain

**Keywords:** catechol, conducting polymers, electropolymerization, gold nanoparticles (AuNPs), polypyrrole

## Abstract

Two different methods were used to obtain polypyrrole/AuNP (Ppy/AuNP) composites. One through the electrooxidation of the pyrrole monomer in the presence of colloidal gold nanoparticles, referred to as trapping method (T), and the second one by electrodeposition of both components from one solution containing the monomer and a gold salt, referred to as cogeneration method (C). In both cases, electrodeposition was carried out through galvanostatic and potentiostatic methods and using platinum (Pt) or stainless steel (SS) as substrates. Scanning electron microscopy (SEM) demonstrated that in all cases gold nanoparticles of similar size were uniformly dispersed in the Ppy matrix. The amount of AuNPs incorporated in the Ppy films was higher when electropolymerization was carried out by chronopotentiometry (CP). Besides, cogeneration method allowed for the incorporation of a higher number of AuNPs than trapping. Impedance experiments demonstrated that the insertion of AuNPs increased the conductivity. As an electrochemical sensor, the Ppy/AuNp deposited on platinum exhibited a strong electrocatalytic activity towards the oxidation of catechol. The effect was higher in films obtained by CP than in films obtained by chronoamperometry (CA). The influence of the method used to introduce the AuNPs (trapping or cogeneration) was not so important. The limits of detection (LOD) were in the range from 10^−5^ to 10^−6^ mol/L. LODs attained using films deposited on platinum were lower due to a synergy between AuNPs and platinum that facilitates the electron transfer, improving the electrocatalytic properties. Such synergistic effects are not so pronounced on stainless steel, but acceptable LOD are attained with lower price sensors.

## Introduction

Polypyrrole (Ppy) is one of the most extensively studied, conducting polymers due to its good electrical conductivity and redox properties [1–2]. Ppy films can be easily generated by electropolymerization and used as a strong adherent layer using different electrochemical techniques [3]. Electrodes that are chemically modified with Ppy have good electrocatalytic activity. For this reason, they have been widely used as chemical sensors for the detection of a variety of substances. The structure and sensing properties of the Ppy films are considerably influenced by the electrochemical method used for the polymerization (potentiostatic, galvanostatic or potentiodynamic), by the electrochemical conditions (such as voltage, intensity, or scan rate), and by other experimental conditions such as the nature and concentration of the doping agent or the nature of the substrate [4]. This versatility can be used to better control the development of electrochemical sensors with the appropriate selectivity, reproducibility and sensibility towards a particular application.

Recently, composite nanomaterials based on conducting polymers and metal nanoparticles (NPs) of different metals have been developed. Gold nanoparticles (AuNPs) have attracted considerable interest because of their unique optical, electronic and catalytic properties [5–8]. Conducting polymer–gold nanoparticle composites exhibit improved physical and chemical properties over their single-component counterparts and are the focus of intensive research [9–12]. In the case of sensors, it has been reported that the insertion of NPs into the sensing layer provides remarkable properties compared to conventional polymeric matrices. Several examples have been reported in the literature. For instance, electrochemically deposited Ppy/AuNP films have demonstrated a great potential to detect DNA [13], ammonia gas at room temperature [14], caffeine [15] or hydroxylamine [16] among others.

Ppy/AuNP composites can be prepared by chemical and electrochemical polymerization. Electrochemical methods provide a better control of the structure and properties of the composite by controlling the electrochemical conditions during film generation [17]. The electrodeposition of the composite can be achieved using different strategies [18], mainly through the electrooxidation of the monomer in the presence of colloidal gold nanoparticles and the corresponding doping agent [19] but also by electrodeposition of polymer and metal from two separate solutions [20–21] or by electrodeposition of both components from one solution containing a monomer and a metal salt [17]. Finally, layers of electrodeposited polypyrrole and gold nanoparticle films can also been obtained from a single solution where PPy chains served as the reductant of tetrachloroauric acid [22].

Most of the works devoted to the electrosynthesis of Ppy/AuNPs films, are often limited to establish recipes to prepare the films and to tests their electrocatalytic or sensing properties. It could be expected that the electrocatalytic and the sensing properties of the Ppy/AuNPs films directly depend on the polymerization conditions. However, the influence of the polymerization conditions in the properties of Ppy/AuNPs electrodes has not been yet studied.

One of the fields where electrochemical sensors are having an important success is in the detection of phenolic compounds, which are strong antioxidant reagents present in foods, with beneficial effects on human health [23]. As phenols are electroactive compounds, they can be detected by amperometric or voltammetric techniques using graphite or platinum electrodes [24–26]. In addition, electrodes chemically modified with a variety of sensing materials (e.g., phthalocyanines or conducting polymers) have been successfully used as voltammetric sensors for the detection of antioxidants [27]. It has also been demonstrated that the combined use of electrocatalytic materials such as phthalocyanines and nanoparticles, can induce synergistic effects that increase the sensitivity of the sensors [28]. Following this idea, Ppy/AuNPs composites could be good candidates as electrocatalytic materials for the detection of phenols.

The objective of this work was to develop new voltammetric sensors based on electrodeposited Ppy/AuNps for the detection of catechol (an antioxidant of interest in the food industry) and to evaluate the influence of the electrodeposition method in their performance. For this purpose Ppy/AuNp films doped with 1-decanesulfonic acid (DSA) were deposited using different methods. The first approach consisted on the electrodeposition of the Ppy/AuNPs films from a solution containing the monomer and tetrachloroauric acid (denoted as “cogeneration”, C). The second approach consisted of the electrodeposition of the Ppy/AuNPs composited from a solution containing the monomer and gold nanoparticles previously formed (denoted as “trapping” method, T). In both methods, electrodeposition was carried out by chronoamperometry (CA) and by chronopotentiometry (CP). Particular attention was paid to the study of the influence of the substrate used for the electrodeposition that was carried out onto classical platinum electrodes and on stainless steel substrates. This aspect could play a crucial role not only in the structure, properties and performance of the sensor but also in the final price.

The structure and sensing properties of voltammetric sensors modified with Ppy/AuNPs films prepared under different conditions were evaluated and compared.

## Results and Discussion

PPy/AuNPs films were prepared using two different approaches referred as “trapping method” and “cogeneration method”, which are described in the Experimental section. The electropolymerization of pyrrole was generated under potentiostatic and galvanostatic conditions on both platinum and stainless steel substrates, resulting in the formation of nanocomposites based on gold nanoparticles within the polypyrrole layer.

### Electropolymerization of Ppy/AuNPs

**Figure 1:**
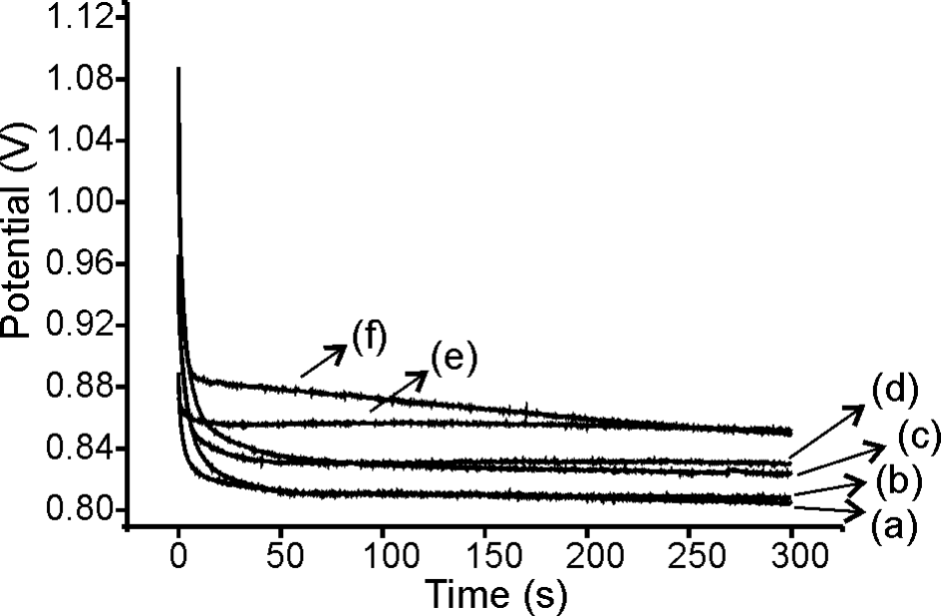
Chronopotentiometric curves obtained during the polymerization of (a) Ppy-CP_Pt_, (b) Ppy-CP_SS_ (c) Ppy/AuNPs-T-CP_SS_ (d) Ppy/AuNPs-T-CP_Pt_, (e) Ppy/AuNPs-C-CP_Pt_ and (f) Ppy/AuNPs-C-CP_SS_.

Figure 1 shows the potential (*E*) vs time (*t*) curves registered during the electrodeposition PPy/AuNPs films using a galvanostatic process. The figure compares the results obtained by the trapping and the cogeneration methods. The CP registered for Ppy (in the absence of AuNPs) is also shown for comparison. As expected, as the current pulse was applied, a sharp decrease in the potential was observed. This was due to the charge of the double layer capacitance that produces a nucleation process at the electrode surface. Then, at the potential at which the monomer is oxidized, a stabilization and growth step was attained, which was characterized by a “plateau”, where the potential varied only slightly.

The highest nucleation rate (faster electrode potential variation) was observed when Ppy was polymerized in the absence of gold nanoparticles or tetrachloroauric acid. At the same time, the final potential (at which the monomer is oxidized) was clearly lower for Ppy films. According to these results, it can be assumed that, the the presence of AuNPs affects the nucleation of Ppy, making impeding the oxidation of the monomers.

The final potential attained when polymerization was carried out in the presence of previously formed AuNPs (trapping), was lower than the potential obtained when AuNPs were generated in situ (cogeneration). This result seems to confirm that AuNPs affect the nucleation process. Only a small difference was found in the final potential attained by Ppy/AuNPs deposited on Pt or on SS.

Nanocomposites Ppy/AuNP were also prepared by trapping and cogeneration using CA. Curves show the characteristic stepped shape of the potentiostatic polymerization: After a short induction period where diffusion controls the monomer oxidation, the current increased rapidly with time, where polymer started nucleating and growing on the electrode surface. Finally, the current reached a plateau coinciding with a continuous and gradual polymer growth [29–30]. The calculated charges are shown in Table 1.

**Table 1:** Polymerization charges calculated for Ppy and Ppy/AuNPs composites prepared by chronoamperometry.

Sample	*Q* (C/cm^2^)	
	SS	Pt

Ppy-CA	0.62	0.62
Ppy/AuNPs-T-CA	0.07	0.08
Ppy/AuNPs-C-CA	0.12	0.22

In good accordance with results shown in previous paragraphs, also when using CA, the polymerization charge was strongly dependent on the presence of AuNPs and the mass deposited in the absence of AuNPs was higher than the mass deposited in the presence of gold. The charge calculated for films obtained by cogeneration was higher than that of the films obtained by trapping. That is, the amount of polymer deposited followed the same trend regardless whether CP or CA was used (Ppy > Ppy/AuNP-C > Ppy/AuNP-T). This result also points to the role of AuNPs in the nucleation of Ppy, which impede the the oxidation of the monomers. The coefficients of variation (% CV) were always lower than 2% regardless of the electropolymerization method or the susbstrate used.

### Structural characterization: SEM studies

**Figure 2:**
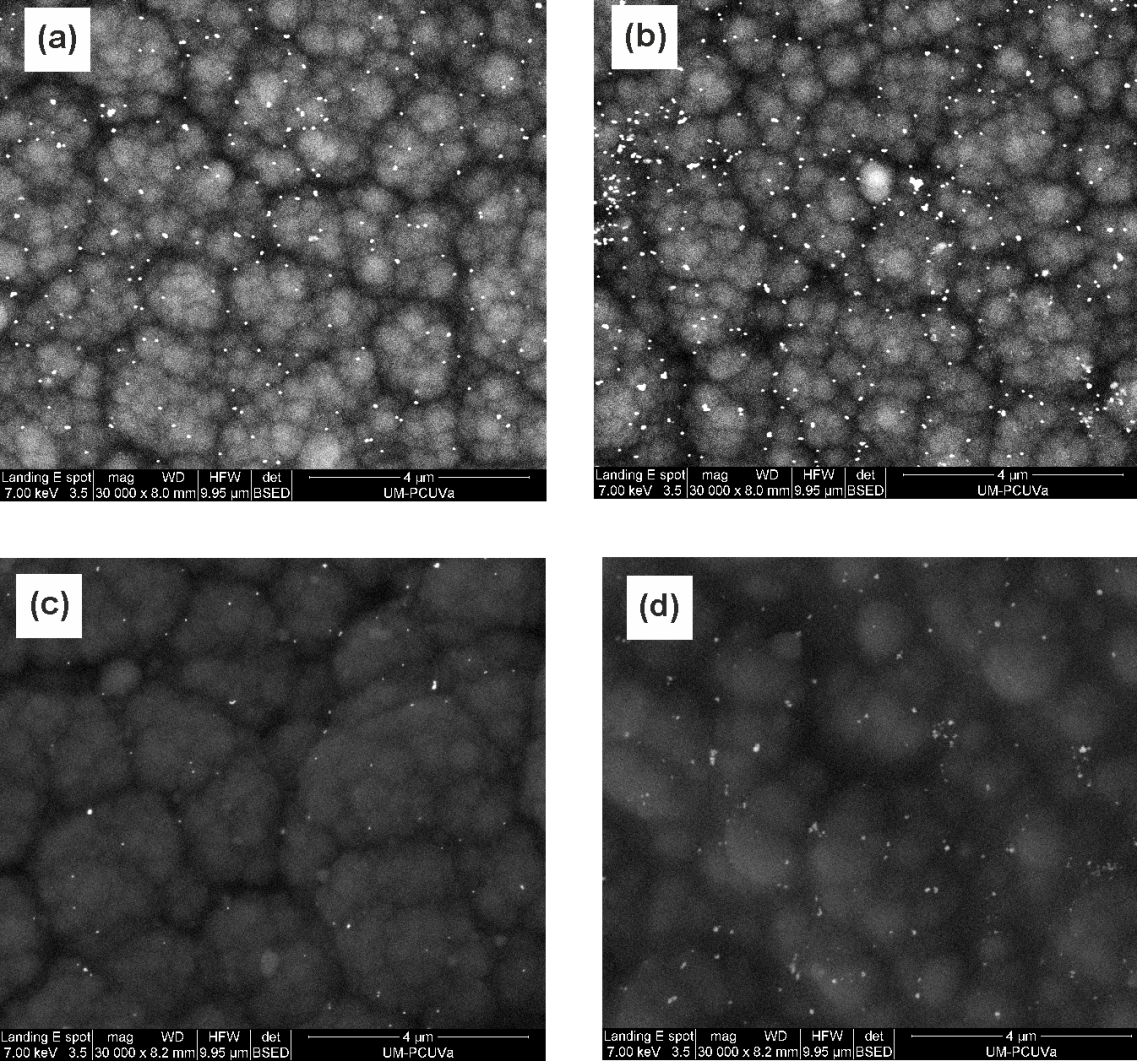
SEM images of Ppy/AuNP fims deposited on SS (a) Ppy/AuNP-T-CPss; (b) Ppy/AuNP-C-CPss; (c) Ppy/AuNP-T-CAss; (d) Ppy/AuNP-C-CAss.

The microscopic structure of the Ppy/AuNP films analyzed by scanning electron microscopy confirmed the incorporation of the AuNPs into the Ppy films (Figure 2). They were uniformly dispersed in the typical granular raspberry PPy matrix. The structures of films deposited onto SS or Pt were almost identical.

The average size of the AuNPs was between 30 and 40 nm (regardless of the method used), which is consistent with the absorbance at 540 nm observed by colloid that was used to obtain the nanocomposites by trapping. The number of AuNPs incorporated in the Ppy films was higher when using CP than that when using CA. In turn, using cogeneration, the amount of nanoparticles incorporated was higher than using trapping.

### Electrochemical impedance spectroscopy

**Figure 3:**
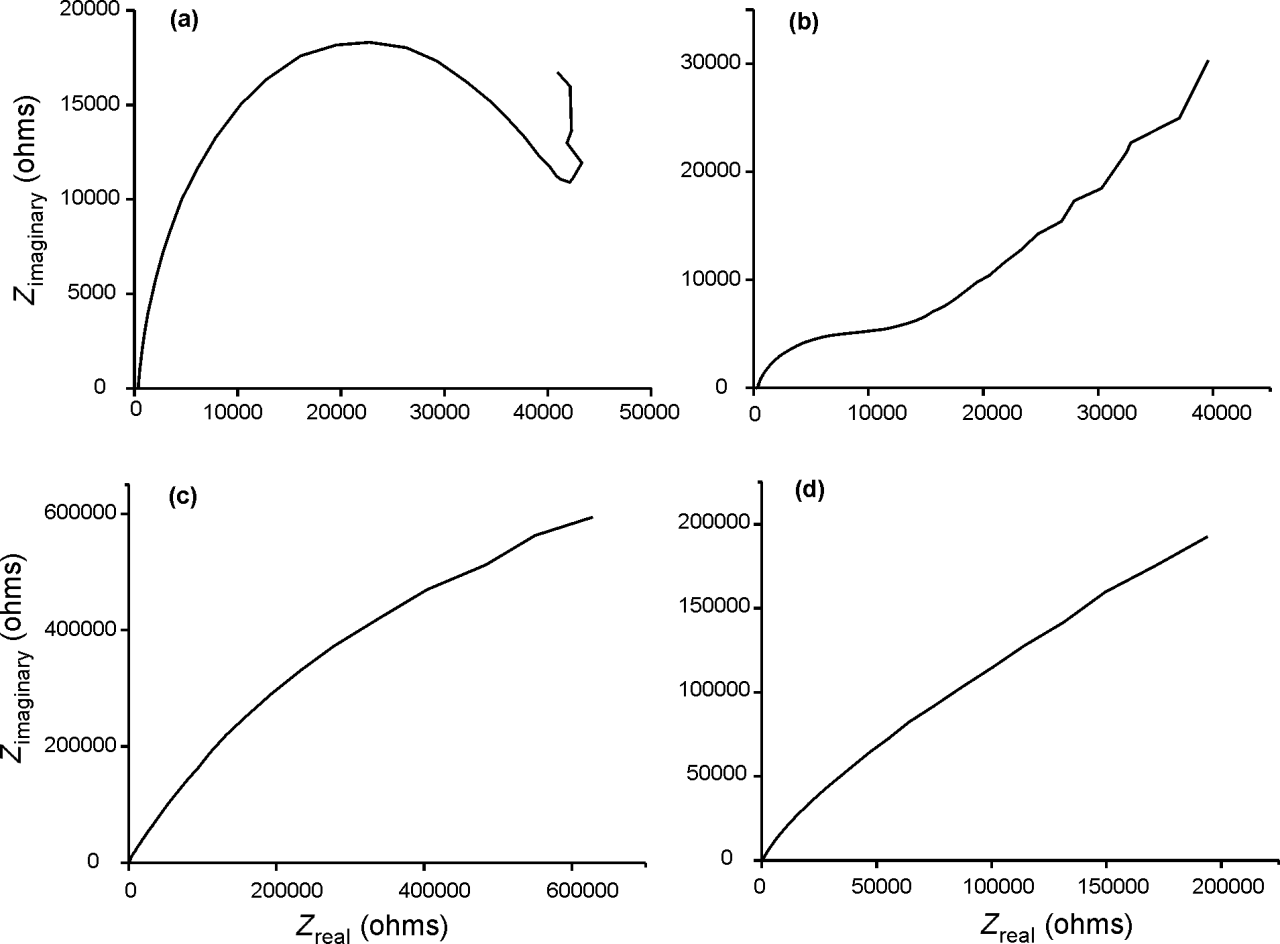
Nyquist plot of films deposited onto Pt registered in 0.1 mol/L KCl. Frequency swept from 105 to 0.1 Hz (a) Ppy-CA_Pt_; (b) Ppy/AuNP-T-CA_Pt_; (c) Ppy/AuNP-C-CA_Pt_; (d) Ppy/AuNP-C-CP_Pt_.

Electrochemical impedance spectroscopy (EIS) can provide information about the conductivity changes resulting from the insertion of AuNPs in the Ppy films. The complex impedance can be plotted as the real (*Z*_real_) vs imaginary (*Z*_imaginary_) components (Nyquist plot), which are related to the resistance and capacitance of the cell, respectively. At high frequencies (left part of the diagram) the semicircular part is associated to electron-transfer limited processes. The diameter of the semicircle is equivalent to the electron-transfer resistance (*R*_ct_). The linear part that appears at lower frequencies is related to diffusion limited processes. In the case of Ppy deposited by CA, the Nyquist plot (Figure 3a) was a semicircle (*R*_ct_, 45.54 kΩ). The electrochemical process was thus, dominated by electron transfer.

The insertion of AuNPs in the Ppy films clearly modified the electrical behavior. In effect, the Nyquist plot of Ppy/AuNP-T-CA_Pt_ films obtained by trapping (Figure 3b) showed a semicircle with a smaller *R*_ct_ (13.52 kΩ) in the high frequencies region. At low frequencies a straight line with a slope of 45° was observed indicating a contribution of both electron transfer and diffusion processes. In Ppy/AuNPs-C-CA_Pt_ films obtained by cogeneration *R*_ct_ was practically zero and only the linear part corresponding to diffusion control was observed (Figure 3c).

These results confirm the ability of AuNPs to reduce the resistance by facilitating the electron transfer. In fact, as observed in SEM images the number of AuNPs inserted in the films was higher using cogeneration, explaining the drastic decrease in the resistance. This is in good agreement with previous published results that indicated that the presence of AuNPs in the polymer matrix resulted in an increase in conductivity [31].

EIS results of Ppy/AuNPs films deposited by CP showed similar trends, but resistance and impedance values were clearly smaller than those observed in films deposited by CA. For instance, the impedance values of Ppy/AuNP-C-CP_Pt_ were one third smaller than those obtained by CA (Figure 3d). Again, the high number of AuNPs inserted in the nanocomposite by CP, explains the improvement in the conductivity.

It is important to point out, that EIS measurements carried out in films deposited on SS by CA where irreproducible, indicating that the films obtained were unstable. Films deposited on SS by CP produced reproducible results but with higher *R*_ct_ and impedance values than those found on the platinum substrate. In fact, in the Nyquist plot for bare Ppy-CP_SS_ the *R*_ct_ was so high that the semicircle was not completed.

According to these results, and taking into account that lower *R*_ct_ values correspond to an increase of the voltammetric signal [32] the cogeneration combined with chronopotentiometry seems to be the most suitable electrodeposition technique to prepare voltammetric sensors.

### Electrochemical behavior of Ppy/AuNPs prepared using different techniques

The electrochemical behavior of Ppy and Ppy/AuNP films was analyzed using cyclic voltammetry in 0.1 mol/L KCl solution. The responses are influenced by the polymerization method, the deposition technique and the type of substrate. Before going into the details, it is important to notice that, in good accordance with previously published results, the first scan was always different from the subsequent cycles. Subsequent cycles were highly reproducible [22]. For this reason, in the next figures, the fifth scan will be displayed.

For Ppy films deposited on platinum using CA or CP, the first cycle showed two redox processes corresponding to the polaron and bipolaron. In successive cycles one single process (anodic wave at −0.35 V and the corresponding cathodic peak at around −0.5 V) was found. When deposition was carried out on SS, voltammograms showed lower intensities and in the case of Ppy-CA_SS_, a certain irreproducibility.

**Figure 4:**
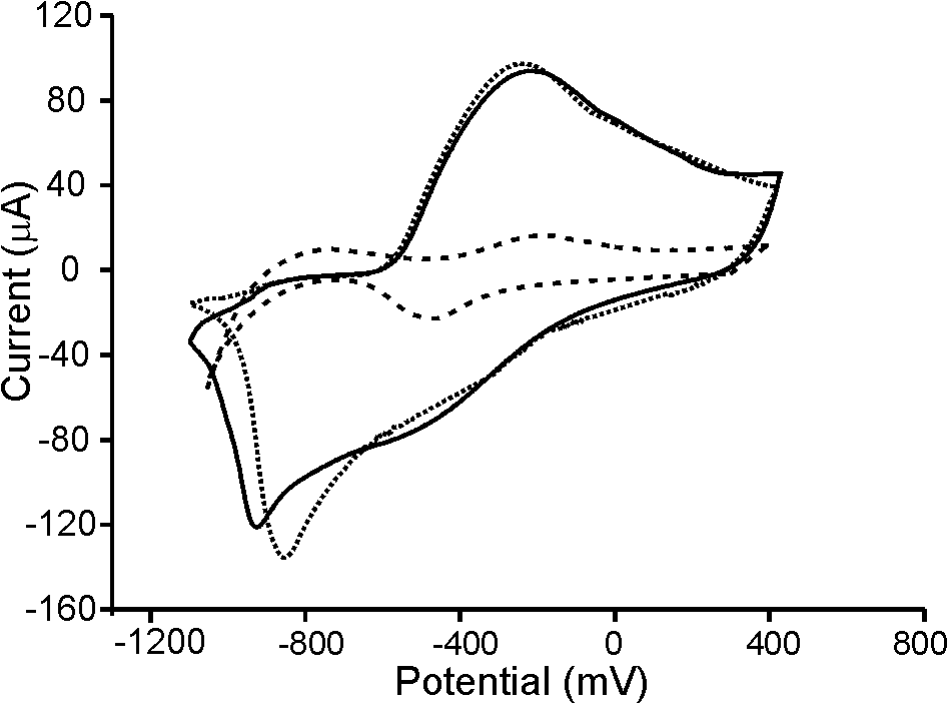
Cyclic voltammograms of Ppy-CP_Pt_ (dashed line), Ppy/AuNPs-T-CP_Pt_ (dotted line) and Ppy/AuNPs-C-CP_Pt_ (solid line) immersed in 0.1 mol/L KCl. Scan rate 0.1 V/s.

When AuNPs were introduced in the films (Ppy/AuNPs), the preparation method induced important differences. In films deposited on platinum, the insertion of AuNPs caused an increase in the intensity of the peaks. Simultaneously the separation between the anodic and the cathodic waves was reduced. This is illustrated in Figure 4 for films deposited on Pt by CP. According to this, it can be concluded that the reversibility of the redox processes is improved in Ppy/AuNP composites. The increase was more pronounced in films deposited by CP than in films deposited by CA. As the number of AuNPs inserted in the films was higher in films deposited by CP (Figure 2), the electrocatalytic effect of the AuNPs is confirmed. This is also in agreement with EIS results that demonstrated that the insertion of AuNPs increased the conductivity.

It is important to point out that, when the deposition was carried out on SS, a decrease in the intensity of the peaks accompanied by a separation between the anodic and cathodic waves was observed. This behavior pointed to the interference between SS and AuNPs. In addition, some irreproducibility was observed (as it also happened in EIS experiments).

In fact, a part from the differences already commented in the electrochemical behavior of Ppy/AuNps films deposited onto Pt and SS, the most remarkable difference was related to their stability and lifetime. We already mentioned that the first cycle was different from the subsequent ones, but the changes occurring in successive cycles were more pronounced in films deposited on stainless steel substrates. The variation coefficients calculated in films deposited on Pt by CP or CA were less than 2% and 5%, respectively. The %CV calculated from films deposited on SS were 8% for CP and 15–20% for CA. Moreover, when electrodes were withdrawn from the solution and reintroduced in the tested solution, electrodes deposited onto SS, changed completely their electrochemical response and could not be further used.

The above results established the important influence of the electropolymerization method (CA or CP) and of the nature of the substrate in the electrochemical properties of the films. The influence of the method used to introduce the AuNPs (trapping or cogeneration) was not so important. In fact, when films were deposited onto Pt, the differences in the voltammograms prepared by trapping or by cogeneration were minimal. In contrast, when SS was used as the substrate, the differences observed between trapping and cogeneration could be ascribed to the irreproducibility and therefore conclusions could not be deduced.

The irreproducibility observed in stainless steel can be clearly attributed to pitting processes produced by chloride ions. In consequence, reproducibility could be improved by changing the supporting electrolyte.

According to this idea, the influence of the supporting electrolyte was further investigated using phosphate buffer. As expected, the large size and high charge of the phosphate anions, made difficult the diffusion of anions inside the polymeric film producing a broadening of the peaks and the increase in the separation between the anodic and the cathodic waves that appeared at −0.15 and −0.8 V, respectively [33] . A part from the broadening of the peaks, the effects caused by AuNPs were similar to those observed in KCl (e.g., increase in the intensity of the peaks accompanied by a decrease in the separation between anodic and cathodic waves.

Using phosphate buffer, the pitting processes were avoided and the reproducibility of films deposited on SS was clearly improved and was similar to that calculated in films deposited on platinum (CV less than 5%).

### Electrocatalytic and sensing behavior towards catechol

Once stable Ppy/AuNP electrodes were obtained, their electrocatalytic and sensing properties towards catechol (a phenolic compound of interest in the food industry), were analyzed in terms of signal amplification and peak shifts. Experiments were carried out in the range between −0.1 and 0.8 V at a scan rate of 0.1 V/s in phosphate buffer. Under these conditions, SS could be used as a substrate due to the absence of pitting processes. Notice also that the polaron–bipolaron response of pyrrole occurs out of this range at negative potentials.

**Figure 5:**
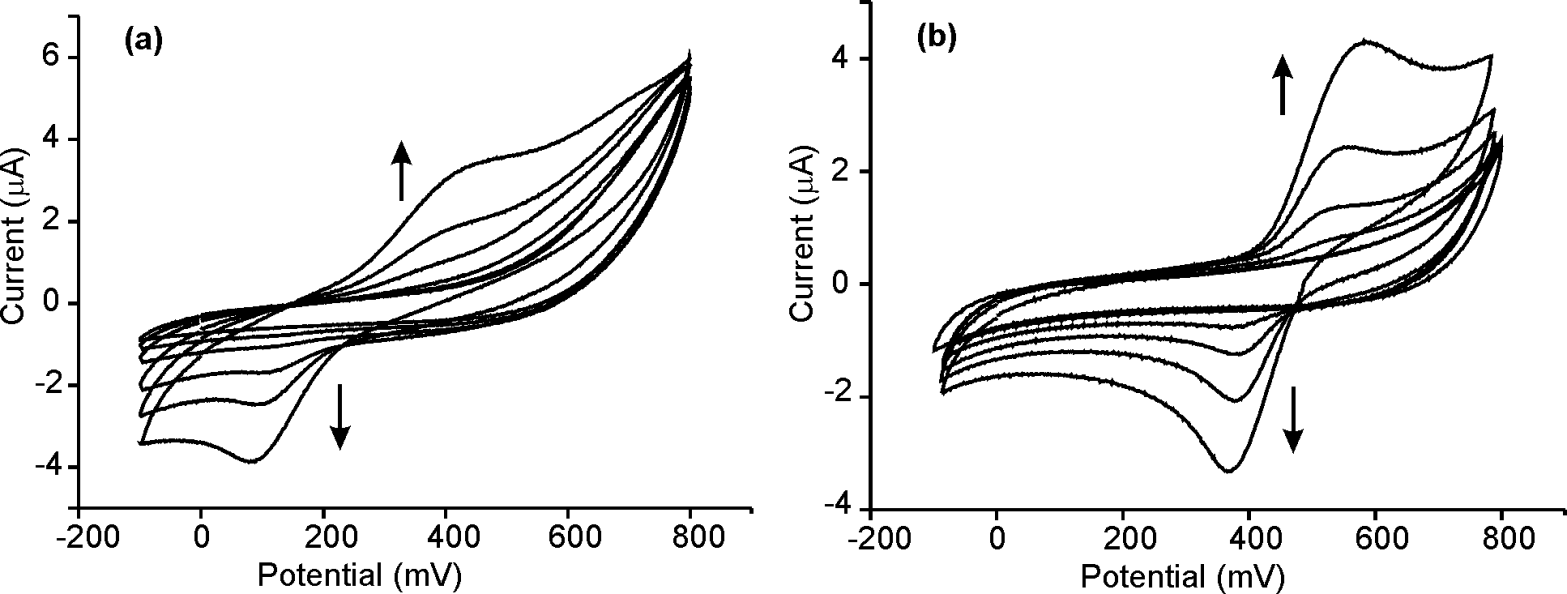
Voltammograms registered using electrodes deposited by CP on SS immersed in 1·10^−5^ to 1·10^−3^ mol/L solutions of catechol in 0.01 mol/L phosphate buffer (pH 7.0): (a) Ppy-CP_SS_ (b) Ppy/AuNP-T-CP_SS_.

Catechol produced the expected well-shaped redox pair generated by the two-electron oxidation/reduction of the *ortho*-dihydroquinone to benzoquinone [26]. The reversibility of the peaks was improved with the incorporation of the AuNPs. Simultaneously, the intensity of the peaks increased with the concentration of AuNPs. This is illustrated in Figure 5 for electrodes deposited on SS by CP. As observed in the Figure, the separation between the anodic and cathodic waves was 300 mV in Ppy-CPss films and only 100 mV in Ppy/AuNP-T-CP_SS_.

These effects were stronger in films deposited by CP than in films deposited by CA, due to the higher concentration of nanoparticles. In contrast, the method to insert the nanoparticles (trapping or cogeneration) only produced small changes in the intensities and positions of the peaks, probably due to the minimal differences in the AuNPs concentration.

The electrocatalytic effect was stronger in films deposited on platinum than in SS. This is in good accordance to previously published reports that have established that AuNPs exhibit a catalytic behavior when deposited onto platinum due to the synergy between both metals [34].

The effect of the concentration of catechol was studied by immersing the electrodes prepared by CP in 1·10^−5^ to 1·10^−3^ mol/L catechol solutions. A linear increase in the intensity of the peaks with the concentration was observed in the studied range. The limit of detection (LOD) was calculated from the calibration curves following the “3sd/m” criterion. As observed in Table 2, the LODs were in the range from 10^−5^ to 10^−6^ mol/L. The LOD obtained using Ppy/AuNP composite films was almost one order of magnitude lower than the one observed in Ppy films. The synergy between platinum and AuNPs increased the sensitivity, allowing a further decrease in the LODs. This synergy is not so important when using SS. Therefore, the use of SS as a substrate, provides stable sensors with good LODs while decreasing the price of the devices considerably.

**Table 2:** LOD, sensitivity and regression coefficients calculated from the anodic and cathodic peaks of catechol.

Sensor	LOD (mol/L) (cathodic peak)	R^2^	LOD (mol/L) (anodic peak)	R^2^

Ppy-CP_Pt_	9.1·10^−5^	0.977	5.3·10^−5^	0.989
Ppy/AuNP-C-CP_Pt_	2.4·10^−5^	0.976	8.8·10^−5^	0.996
Ppy/AuNP-T-CP_Pt_	0.9·10^−5^	0.984	0.3·10^−5^	0.981
Ppy-CP_SS_	8.9·10^−5^	0.956	7.2·10^−5^	0.975
Ppy/AuNP-C-CP_SS_	4.3·10^−5^	0.977	3.1·10^−5^	0.971
Ppy/AuNP-T-CP_SS_	3.2·10^−5^	0.968	1.1·10^−5^	0.975

## Conclusion

Ppy/AuNP nanocomposites have been successfully prepared employing in situ polymerization of pyrrole using tetrachloroauric acid as an oxidant in the presence of gold ions and by trapping AuNPs in a Ppy matrix during the electropolymerization. SEM images confirmed the formation of uniform nanocomposites on smooth platinum and stainless steel substrates.

The presence of AuNPs in the polymer matrix resulted in an increase in the conductivity and in the intensity of the voltammetric signals. These variations in conductivity and intensity of voltammograms are directly related to the number of AuNPs inserted in the Ppy films.

Irreproducibility observed in the EIS and voltammetric measurements carried out in KCl using films deposited on stainless steel, caused by pitting process can be avoided by using phosphate buffer as supporting electrolyte.

As an electrochemical sensor, the Ppy/AuNP deposited on platinum exhibited important electrocatalytic activity towards the oxidation of catechol. The effect was higher in films obtained by CP than in films obtained by CA. The influence of the method used to introduce the AuNPs (trapping or cogeneration) was not so important. The detection limits were in the range of 10^−5^ to 10^−6^ mol/L, which is lower than the concentration usually found in foods and beverages such as wines and musts. The synergy between Pt and Au nanoparticles gave rise to lower LODs. In turn, stainless steel can be used as the substrate in the absence of KCl, with a LOD only slightly higher than those obtained in sensors deposited on Pt, but at a lower cost.

## Experimental

### Reagents and solutions

All experiments were carried out in deionized Milli-Q water (Millipore, Bedford, MA). Pyrrole, tetrachloroauric acid, 1-decanesulfonic acid (DSA), potassium chloride, sodium phosphate, potassium phosphate and catechol were purchased from Sigma-Aldrich. Commercially available reagents and solvents were used without further purification.10^−3^ mol/L stock solutions of catechol were prepared by solving the corresponding compound in KCl solution (0.1 mol/L) or phosphate buffer solution (pH 7.0; 0.1 mol/L). Solutions with lower concentration were prepared from the stock solutions by dilution.

### Preparation of the Au colloidal suspension

The synthesis of AuNPs colloids was carried out according to the procedure proposed by Slot and Geuze [35]. Two solutions were prepared: (1) HAuCl_4_ (0.25·10^−3^ mol/L) in deionized water and (2) sodium citrate dehydrate (17·10^−3^ mol/L) in deionized water. 20 mL of solution (1) was heated until boiling on a hot plate, then 1 mL of solution (2) was quickly added to the HAuCl_4_ solution while stirring. The mixture was then boiled for 20 min. Using this procedure, a red colloid with a UV absorbance maximum at λ = 540 nm was obtained.

### Instruments

Electropolymerizations and electrochemical studies were carried out at room temperature in an EG&G Parstat 2273 potentiostat/galvanostat using a three-electrode configuration. The same instrument was used for the EIS experiments. UV–vis spectra were recorded on a Shimadzu UV-2600 model spectrometer. A SEM-FEI (QUANTA 200F) was used to record the images of the electrode surfaces.

### Electropolymerization methods

The auxiliary electrode was a conventional Pt electrode. The reference electrode was an Ag/AgCl electrode in a 3 mol/L KCl solution. Pt and stainless steel 316L (SS) disks (1 mm diameter) were used as working electrodes. The disks were polished with 0.3 µm alumina suspension using a microcloth polishing pad and rinsed with deionized water in an ultrasonic bath.

#### Electropolymerization of Ppy films

The Ppy films were obtained by electropolymerization from a solution containing 0.1 mol/L pyrrole and 0.05 mol/L 1-decanesulfonic acid (DSA) using two electrochemical techniques: chronopotentiometry (CP) using a constant potential at 0.8 V over a period of 300 s, and chronoamperometry (CA) using 0.02 mA over a period of 300 s (except otherwise indicated). Films were deposited onto Pt and SS.

#### Electropolymerization of Ppy/AuNPs films

Ppy/AuNPs films were obtained using two different approaches. On one hand, Ppy/AuNPs films were synthesized by the “trapping method” from a solution containing 0.2 mol/L pyrrole, 0.1 mol/L DSA. This solution was mixed (1:1) with a solution containing AuNPs previously formed (Au colloidal suspension). Films were polymerized by chronoamperometry using a constant potential at 0.8 V over a period of 300 s, and by chronopotentiometry using 0.02 mA over a period of 300 s. Sensors obtained by trapping were termed as Ppy/AuNP-T-CA (obtained by chronoamperometry) and Ppy/AuNP-T-CP (obtained by chronopotentiometry).

Ppy/AuNPs films were also synthesized using the “cogeneration method” by mixing a solution containing tetrachloroauric acid 10^−3^ mol/L and a solution containing pyrrole and DSA. In this method, and according to the oxidation potentials of pyrrole (0.7 V vs SCE) and the reduction potential of AuCl_4_^−^ (1 V), the AuNPs where generated in situ and inserted in the polymeric film during the electrochemical growth. Also in this case, electropolymerization was carried out by CA and CP under the same conditions used for trapping. Sensors obtained by cogeneration were termed as Ppy/AuNP-C-CA (obtained by chronoamperometry) and Ppy/AuNP-C-CP (obtained by chronopotentiometry).

In all cases, films were deposited onto Pt and SS disks. The type of substrate will be denoted using a subscript (i.e., Ppy/AuNPs-C-CP_Pt_ or Ppy/AuNPs-T-CP_SS_). Once prepared, the polymeric films were extracted from the generation solution and washed thoroughly with water.

### Electrochemical impedance spectroscopy (EIS) characterization

EIS was performed in a 0.1 mol/L KCl solution with a frequency range from 105 to 0.1 Hz and a signal amplitude of 10 mV, at a working potential of 0.0 V.

### Tests of the voltammetric sensors

The Ppy and Ppy/AuNPs films were used as working electrodes in electrochemical experiments. The reference electrode was Ag/AgCl/KCl 3 mol/L and the counter electrode was a platinum wire.

Cyclic voltammetry was carried out at room temperature with a scan rate of 0.1 V/s in the potential range between −1.0 V and 0.8 V (vs Ag/AgCl) except otherwise indicated.

Calibration curves were constructed from catechol solutions with concentrations ranging from 1·10^−5^ to 1·10^−3^ mol/L. The limits of detection (LODs) were calculated following the “3sd/m” criterion, where “m” is the slope of the calibration graph, and “sd” was estimated as the standard deviation (*n* = 5) of the voltammetric signals at the concentration level corresponding to the lowest concentration of the calibration plot [36–37].
